# BET activity plays an essential role in control of stem cell attributes in *Xenopus*

**DOI:** 10.1242/dev.202990

**Published:** 2024-07-03

**Authors:** Paul B. Huber, Anjali Rao, Carole LaBonne

**Affiliations:** ^1^ Department of Molecular Biosciences, Northwestern University, Evanston, IL 60208, USA; ^2^ National Institute for Theory and Mathematics in Biology, Northwestern University, Evanston, IL 60208, USA

**Keywords:** Bromodomain, Brd4, Histone Acetylation, Pluripotency, Stem cell, *Xenopus*, Neural crest

## Abstract

Neural crest cells are a stem cell population unique to vertebrate embryos that retains broad multi-germ layer developmental potential through neurulation. Much remains to be learned about the genetic and epigenetic mechanisms that control the potency of neural crest cells. Here, we examine the role that epigenetic readers of the BET (bromodomain and extra terminal) family play in controlling the potential of pluripotent blastula and neural crest cells. We find that inhibiting BET activity leads to loss of pluripotency at blastula stages and a loss of neural crest at neurula stages. We compare the effects of HDAC (an eraser of acetylation marks) and BET (a reader of acetylation) inhibition and find that they lead to similar cellular outcomes through distinct effects on the transcriptome. Interestingly, loss of BET activity in cells undergoing lineage restriction is coupled to increased expression of genes linked to pluripotency and prolongs the competence of initially pluripotent cells to transit to a neural progenitor state. Together these findings advance our understanding of the epigenetic control of pluripotency and the formation of the vertebrate neural crest.

## Teaser

**Highlighted Article:** BET-family epigenetic readers are essential regulators of the developmental potential of both pluripotent blastula and neural crest cells.

## INTRODUCTION

The neural crest is a cell population unique to vertebrates which is distinguished by its broad multi-germ layer developmental potential (Le Douarin and Dupin, 2014). These cells have the ability to contribute to a diverse array of highly specialized cell types which are concomitant with vertebrate-specific features, including bone and cartilage of the craniofacial skeleton, sensory neurons and glia of the peripheral nervous system, and pigment-producing melanocytes (Martik and Bronner, 2017; Simões-Costa and Bronner, 2015; Soldatov et al., 2019; York and McCauley, 2020). Understanding the origins and development of neural crest cells, and in particular their stem cell attributes, is essential to understanding the early evolution of vertebrates.

Recent insights into the origins of neural crest cells arose from the observation that much of the transcriptional circuitry that controls their developmental potential is shared with blastula-stage pluripotent cells (Buitrago-Delgado et al., 2015; Lavial et al., 2007; Lignell et al., 2017; Lukoseviciute et al., 2018; Scerbo and Monsoro-Burq, 2020; Schock et al., 2022; Zalc et al., 2021). Transcription factors Snail1, Sox5, FoxD3, Myc and TFAP2 have been shown to play roles in the formation of neural crest and the maintenance of pluripotency in blastula stem cells (Schock et al., 2022). These findings suggest a model in which neural crest cells arose via retention of characteristics of those earlier cells. Consistent with such a model, a requirement for BMP signaling and FGF-mediated MAP kinase signaling have also been found to be shared attributes of pluripotent blastula cells and neural crest cells in *Xenopus* (Geary and LaBonne, 2018; Nordin and LaBonne, 2014). Both cell types are also characterized by low levels of histone acetylation and a requirement for histone deacetylase (HDAC) activity, indicating the important, yet less well studied, roles that epigenetic factors play in these cells (Rao and LaBonne, 2018).

A growing body of work indicates that the regulation of histone acetylation plays a key role in the establishment of the neural crest and the maintenance of stem cell pluripotency (Bogdanović et al., 2012; Esmaeili et al., 2020; Feng et al., 2022; Hezroni et al., 2011; Hu et al., 2014; Karmodiya et al., 2012; Kong et al., 2014; Rao and LaBonne, 2018; Schwenty-Lara et al., 2020; White et al., 2021). HDACs, which act as ‘erasers’ to remove acetyl marks, play a crucial role in the maintenance of pluripotency in cultured embryonic stem cells (ESCs) (Dovey et al., 2010; Jamaladdin et al., 2014; Nieto-Estevez et al., 2021; Sun et al., 2011). In *Xenopus*, inhibition of HDAC activity leads to a loss of blastula-stage pluripotency and neural crest, whereas increasing HDAC1 activity was found to enhance reprogramming to a neural crest cell state (Rao and LaBonne, 2018). If control of histone acetylation is important for pluripotency and neural crest development, a key question is what recognizes these acetylation marks and what consequence that has for gene expression during these key stages of development.

BET (bromodomain and extra-terminal) proteins are a family of epigenetic ‘readers’ of acetylated histones and proteins that translate the state of chromatin into gene expression by recruiting key transcriptional regulatory factors, including TATA-binding protein and the IRF3/p300 and p-TEFb complexes, to their docking sites (Hargreaves et al., 2009; McClure et al., 2013; Peng et al., 2007; Ren et al., 2017). BET proteins, which includes somatic members BRD2, BRD3, BRD4 and the testis-specific BRDT, possess two adjacent bromodomains that bind acetylated lysine residues on histones and other proteins to facilitate transcriptional activation (Wu and Chiang, 2007). Unique to BRD4 is an extended C-terminal region of ∼600 amino acids, and this results in additional functions of BRD4 compared with other BET members, most notably the recruitment of P-TEFb (Hsu and Blobel, 2017). BRD2, BRD3 and BRD4 bind both overlapping as well as distinct places in the genome (Dai et al., 2019; Lotke et al., 2020; Trivedi et al., 2020). Their distinct roles during embryogenesis remain unclear.

Of the BET proteins, only BRD4 has thus far been linked to the regulation of pluripotency or neural crest development (Fernandez-Alonso et al., 2017; Linares-Saldana et al., 2021; Wu and Donohoe, 2015; Zhang et al., 2022). BRD4 has been found to interact with OCT4 in ESCs and can influence regulation of the Nanog promoter (Horne et al., 2015; Wu et al., 2015). BRD4 is essential for post-implantation development in mice and for pluripotency in cultured ESCs, highlighting the importance of BRD4 as a reader of acetylation marks (Gonzales-Cope et al., 2016; Houzelstein et al., 2002; Shi and Vakoc, 2014). More recently, BET protein activity was shown to be required for the specification and maintenance of the epiblast lineage in mice, and BRD4 specifically was shown to activate multiple pluripotency-associated super-enhancers in three mammalian species (Tsume-Kajioka et al., 2022; Zhang et al., 2022).

In this study, we asked whether BET proteins/BRD4 are necessary for the regulation of stem cell attributes in pluripotent blastula or neural crest cells in *Xenopus* to better understand how their roles are conserved or differ across the vertebrate clade, and the extent to which BET activity highlights similar epigenetic mechanisms shared between these two stem cell populations. Here, we report that BET activity is essential for both maintenance of pluripotency in naïve blastula cells and the formation of neural crest cells, similar to HDAC activity (Rao and LaBonne, 2018). We compared the effects of HDAC or BET inhibition on the transcriptome and found that, whereas HDAC inhibition leads to the upregulation of genes which direct multiple lineage states, BET inhibition largely leads to downregulation of target genes at blastula stages. However, in lineage-restricted blastula explants, loss of BET activity leads to an upregulation of pluripotency and neural gene expression, indicating that those cells do not properly exit the pluripotent state and adopt neural features. Moreover, inhibition of BET/BRD4 activity prolongs the competence of initially pluripotent cells to transit to a neural progenitor state.

## RESULTS

### BET activity is required for establishment of neural crest

**Fig. 1.**
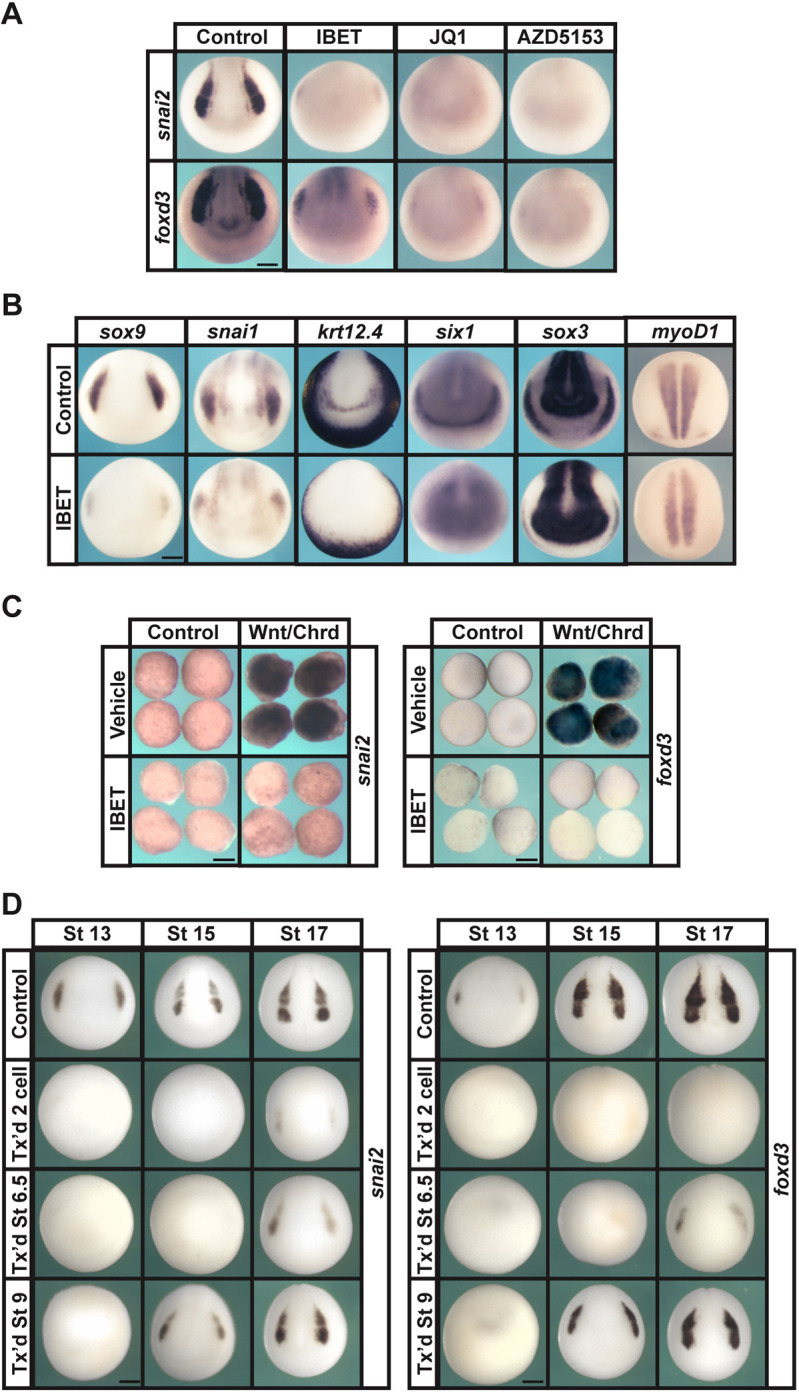
**BET activity is required for neural crest formation.** (A) *In situ* hybridization examining the expression of neural crest factors *snai2* and *foxd3* following treatment with vehicle or inhibitor (250 µM IBET, 10 µM JQ1, 100 µM AZD5153). Embryos were collected at stage 15 (mid neurula). (B) *In situ* hybridization examining expression of neural crest factors *sox9* and *snai1*, epidermal marker *krt12.4*, mesodermal marker *myoD1*, placodal marker *six1* and neural plate marker *sox3* following treatment with vehicle or IBET. Embryos were treated at two-cell stage and collected at stage 15 (mid neurula). (C) *In situ* hybridization examining expression of *snai2* and *foxd3* in Wnt/Chrd-induced explants treated with vehicle or IBET. Explants were collected at stage 18 (late neurula). (D) *In situ* hybridization examining expression of neural crest factors *snai2* and *foxd3* following treatment with vehicle or IBET at the indicated stage (two-cell, 6.5 or 9). Embryos were collected at stages 13, 15 or 17 (early, mid or late neurula, respectively). Scale bars: 250 μm.

To determine whether BET protein activity is required for neural crest formation, we treated two-cell-stage *Xenopus laevis* embryos with three different small molecule inhibitors – IBET (I-BET762), JQ1 and AZD5153, all of which specifically block the binding of BET bromodomains to acetylated lysine residues on histones (Filippakopoulos et al., 2010; Mirguet et al., 2013; Rhyasen et al., 2016) – or with vehicle. Treatment with any of these inhibitors resulted in either complete loss or severe reduction of the neural crest markers *snai1* (IBET: 100%, *n*=85; DMSO: 0%, *n*=86), *snai2* (IBET: 100%, *n*=96; JQ1: 100%, *n*=52; AZD5153: 100%, *n*=46; DMSO: 0%, *n*=39), *sox9* (IBET: 100%, *n*=110; DMSO: 0%, *n*=104) and *foxd3* (IBET: 100%, *n*=106; JQ1: 100%, *n*=46; AZD5153: 100%, *n*=38; DMSO: 0%, *n*=39) at neurula stages (Fig. 1A,B). Loss of neural crest continued to be observed through early tadpole stages, and residual neural crest at these stages did not migrate properly, as evidenced by expression of *tfap2a* [stage (st.) 23 (IBET: 100%, *n*=42; DMSO: 0%, *n*=47), st. 25 (IBET: 100%, *n*=45; DMSO: 0%, *n*=39)] and *twist1* [st. 23 (IBET: 100%, *n*=53; DMSO: 0%, *n*=46), st. 25 (IBET: 100%, *n*=39; DMSO: 0%, *n*=38)] (Fig. S1A). IBET treatment at the two-cell stage also resulted in a dorsal reduction in epidermal keratin (*krt12.4*) (IBET: 100%, *n*=108; DMSO: 0%, *n*=106) and loss of the placodal marker *six1* (IBET: 100%, *n*=93; DMSO: 0%, *n*=90) (Fig. 1B). The expression of *sox3* in placodal regions was lost in IBET-treated embryos, although the *sox3* expression in the neural plate was expanded (IBET, *n*=106; DMSO, *n*=115) (Fig. 1B; Fig. S1B). We also observed a decrease in expression of a marker of somitic mesoderm, *myoD* (also known as *myoD1*), However, this was a less dramatic decrease than that observed for ectodermal tissues (IBET, *n*=80; DMSO, *n*=79).

Given the strong loss of neural crest in response to BET inhibition, we asked whether BET activity was also required for programming animal pole explants to a neural crest state. Embryos were injected with mRNA encoding Wnt8 and the BMP antagonist chordin (Chrd) at the two-cell stage and cultured to blastula stages, when animal pole cells were explanted. Control and injected explants were then treated with IBET or vehicle (DMSO) and cultured to mid-neurula stages, when explants were collected and expression of neural crest markers *snai2* and *foxd3* was examined. IBET was found to strongly inhibit expression of both of these markers [*snai2* (IBET: 100%, *n*=30; DMSO: 3%, *n*=32), *foxd3* (IBET: 100%, *n*=31; DMSO: 3%, *n*=31)], further confirming that the activity of one or all of the members of the BET family is involved in the establishment of the neural crest progenitor state (Fig. 1C).

To better determine when BET activity is required during neural crest formation, we treated embryos with IBET or vehicle at the two-cell stage, around the time of the onset of zygotic transcription (stage 6.5), or at late blastula stage (stage 9) (Fig. 1D). We found that inhibition of BET activity beginning at the two-cell stage led to loss of neural crest markers through stage 17 [*snai2* (IBET: st. 13 100%, *n*=61; st. 15 100%, *n*=74; st. 17 100%, *n*=79; DMSO: st. 13 0%, *n*=50; st. 15 0%, *n*=74; st. 17 0%, *n*=74), *foxd3* (IBET: st. 13 100%, *n*=64; st. 15 100%, *n*=68; st. 17 100%, *n*=77; DMSO: st.13 0%, *n*=68; st. 15 0%, *n*=76; st. 17 0%, *n*=68)]. When IBET treatment was initiated at stage 6.5 these markers were inhibited through mid-neurula stages, however all embryos showed some recovery of *snai2* and *foxd3* by stage 17 [*snai2* (st. 13 100%, *n*=86; st. 15 100%, *n*=72; st. 17 100%, *n*=74), *foxd3* (st. 13 100%, *n*=76; st. 15 100%, *n*=74; sta. 17 100%, *n*=79)]. Inhibiting BET activity starting at late blastula stages led to loss of *snai2* and *foxd3* expression at stage 13, but recovery of some *snai2* and *foxd3* was evident in all embryos starting at stage 15 [*snai2* (st. 13 100%, *n*=51; st. 15 100%, *n*=48; st. 17 100%, *n*=43), *foxd3* (st. 13 100%, *n*=52; st. 15 100%, *n*=48; st. 17 100%, *n*=47)]. Collectively, these findings suggest that the most crucial period for BET activity during neural crest genesis is around the mid-blastula stage, when pluripotent blastula cells arise.

### BET activity is required for pluripotency gene expression and lineage restriction

**Fig. 2.**
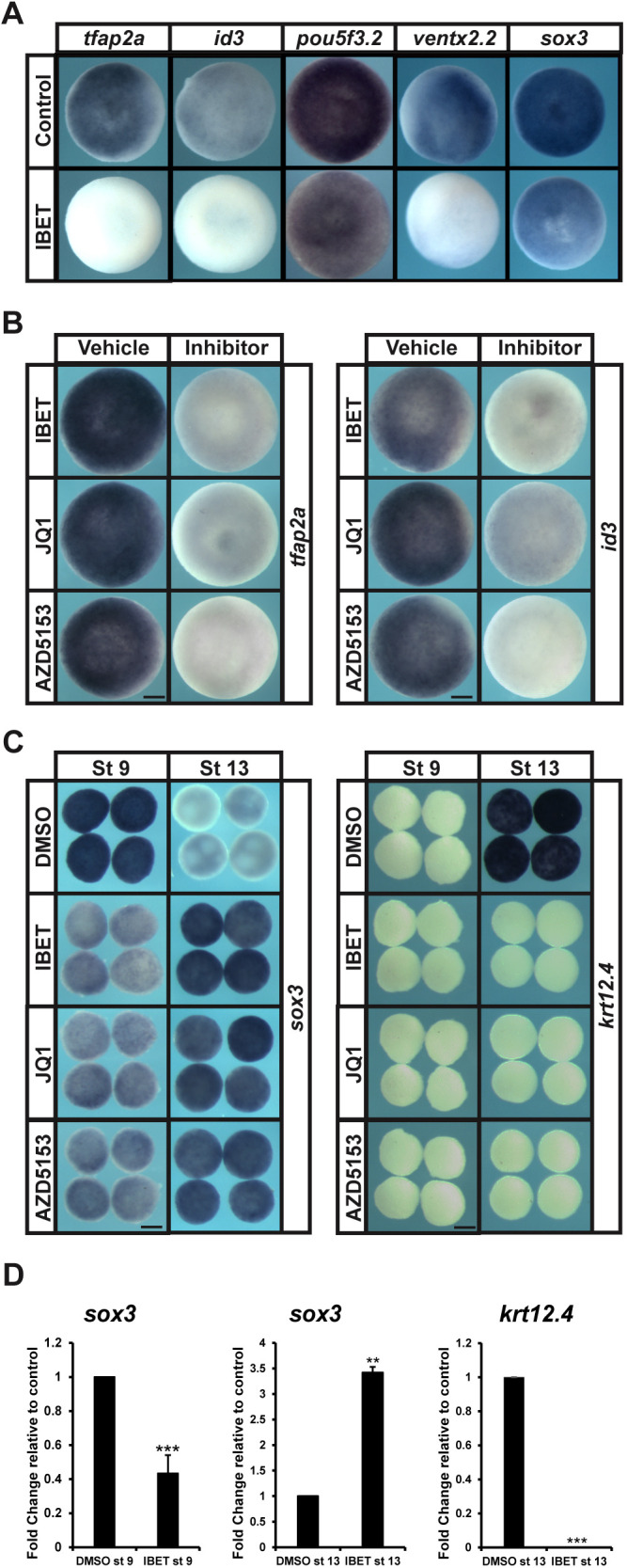
**BET activity is essential for expression of pluripotency genes and proper lineage restriction.** (A) *In situ* hybridization examining expression of *tfap2a*, *id3*, *pou5f3.2*, *ventx2.2* and *sox3* in pluripotent blastula cells following treatment with vehicle or IBET (250 µM). Embryos were treated at the two-cell stage and collected at stage 9 (late blastula). (B) *In situ* hybridization examining expression of *tfap2a* or *id3* in pluripotent blastula cells following treatment with vehicle or inhibitor (250 µM IBET, 10 µM JQ1, 100 µM AZD5153). Embryos were treated at the two-cell stage and collected at stage 9 (late blastula). (C) *In situ* hybridization examining expression of *sox3* and *krt12.4* in explanted blastula caps treated with vehicle or IBET. Embryos were treated at the two-cell stage, then explants were grown in culture with sibling embryos for staging purposes and collected at stage 9 (late blastula) and stage 13 (early neurula). (D) qRT-PCR of explanted blastula caps examining expression of *sox3* or *krt12.4* following treatment with vehicle or IBET (250 µM). **P<0.01, ***P<0.001 (standard two-tailed *t*-test with two sample equal variance). Data are mean±s.e.m. Explants were treated at the two-cell stage, then explants were grown in culture with sibling embryos and collected at stage 9 (late blastula) and stage 13 (early neurula). Scale bars: 250 μm.

If BET activity is required to establish blastula-stage pluripotency, then blocking BET activity might be expected to alter the expression of key regulators of this state. To determine this, embryos treated with IBET at the two-cell stage were examined at blastula stages for expression of pluripotency factors *pou5f3.2* (Morrison and Brinkman, 2006), *sox3* (Gentsch et al., 2019), *vent2* (*ventx2.2*; functional ortholog of *nanog*; Scerbo et al., 2012), *tfap2a* (Driscoll et al., 2024) and *id3* (Jiang et al., 2022), compared with DMSO-treated control embryos (Fig. 2A).

Expression of all of these genes was downregulated in response to IBET in all embryos, although the degree of downregulation was most dramatic for *tfap2a* and *id3* [*pou5f3.2* (IBET: 100%, *n*=77; DMSO: 0%, *n*=67), *sox3* (IBET: 100%, *n*=80; DMSO: 0%, *n*=85), *ventx2.2* (IBET: 100%, *n*=50; DMSO: 0%, *n*=66), *tfap2a* (IBET: 100%, *n*=98; DMSO: 0%, *n*=93), *id3* (IBET: 100%, *n*=70; DMSO: 0%, *n*=69)]. Complete loss of *tfap2a*, *id3*, *ventx2.2* and *sox3* was also seen with JQ1 and AZD5153, demonstrating the specificity of these inhibitors for BET protein activity [*tfap2a* (JQ1: 100%, *n*=82; AZD5153: 100%, *n*=91; DMSO: 0%, *n*=73), *id3* (JQ1: 100%, *n*=49; AZD5153: 100%, *n*=82; DMSO: 0%, *n*=85), *ventx2.2* (JQ1: 100%, *n*=82; AZD5153: 100%, *n*=91; DMSO: 0%, *n*=87), *sox3* (JQ1: 100%, *n*=49; AZD5153: 100%, *n*=82; DMSO: 0%, *n*=68)] (Fig. 2B; Fig. S2A).

Given that BET activity is required for expression of pluripotency markers during blastula stages, we next asked how BET inhibition would impact the ability of blastula stem cells to progress from a pluripotent (stage 9) to a lineage-restricted (stage 13) state. Pluripotent blastula explants that receive no alternative instruction will adopt an epidermal fate due to intrinsic BMP activity (Fig. 2C). We found that inhibition of BET activity with IBET, JQ1 or AZD5153 from the two-cell stage resulted in a strong decrease of pluripotency marker *sox3* at stage 9, consistent with whole embryo observations (IBET: 100%, *n*=48; JQ1: 100%, *n*=27; AZD5153: 100%, *n*=28; DMSO: 0%, *n*=56). However, at stage 13, *sox3* was upregulated in treated explants compared with controls (IBET: 100%, *n*=51; JQ1: 100%, *n*=30; AZD5153: 100%, *n*=26; DMSO: 0%, *n*=50) and these explants failed to form epidermis, as evidenced by lack of *krt12.4* expression (IBET: 100%, *n*=52; JQ1: 100%, *n*=25; AZD5153: 100%, *n*=31; DMSO: 0%, *n*=52) (Fig. 2C). These findings were confirmed and quantified by qPCR (Fig. 2D). As this result could also be interpreted as a switch from an epidermal to a neural fate, we asked whether other pluripotency factors were affected by IBET in a similar manner. RNA-seq and differential expression analysis (DESeq2) of RNA isolated from blastula explants (at stages 9 and 13) after IBET or vehicle treatment showed that the expression of other core Yamanaka factors, *pou5f3.2* and *ventx2.2*, was significantly decreased at stage 9 and increased at stage 13, similar to *sox3*, further suggesting that BET activity is required for pluripotency in blastula explants (Fig. S2B).

### BET activity is required for blastula-stage pluripotency

**Fig. 3.**
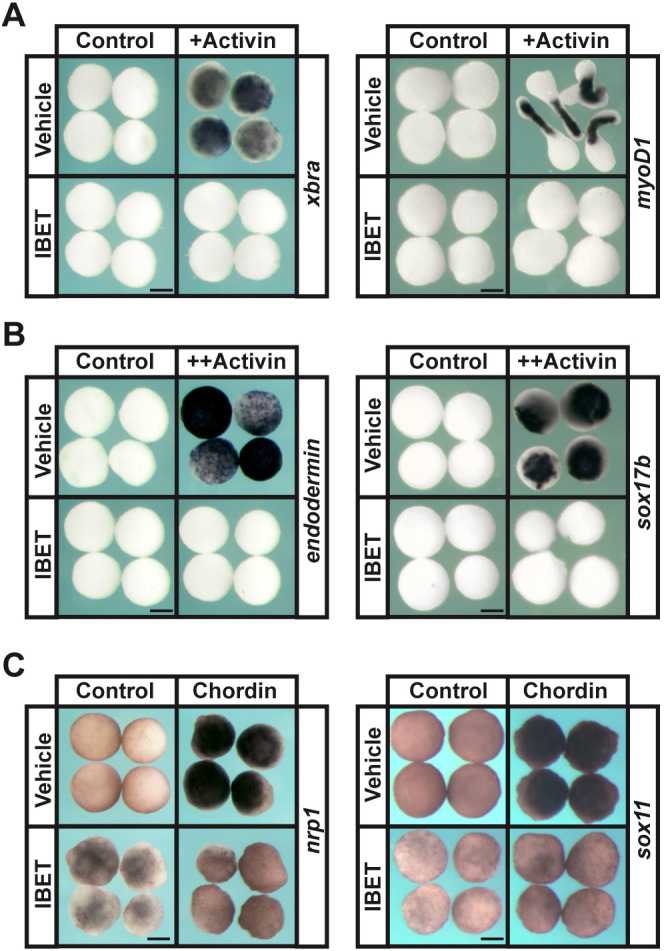
**BET activity is required for the pluripotency of blastula stem cells.** (A,B) *In situ* hybridization examining expression of mesodermal markers (*xbra* and *myoD1*; A) and endodermal markers (*endodermin* and *sox17b*; B) in blastula explants induced with activin and treated with vehicle or IBET (250 µM). Embryos were treated at the two-cell stage, then explants were grown in culture with sibling embryos for staging purposes and collected at stage 11.5 (mid-gastrula) for *xbra*, *endodermin* and *sox17b* expression and stage 18 (late neurula) for *myoD1* expression. (C) *In situ* hybridization examining expression of neural markers (*nrp1* and *sox11*) in blastula explants induced with *chrd* mRNA and treated with vehicle or IBET. Embryos were treated at the two-cell stage and explants were collected at stage 18 (late neurula). Scale bars: 250 μm.

To further examine a role for BET activity in establishing blastula-stage pluripotency, we challenged blastula explants with instructional cues to adopt mesoderm, endoderm and neural fates. Control explants treated with low doses of activin will give rise to mesoderm, as evidenced by expression of *xbra* (*tbxt*) at stage 11.5 and *myoD1* at stage 18, but when BET activity is inhibited, expression of these markers is lost [*xbra* (IBET: 97%, *n*=31; DMSO: 6.8%, *n*=29), *myoD1* (IBET: 100%, *n*=36; DMSO: 9.4%, *n*=32)] and late stage explants fail to elongate (Fig. 3A)*.* Similarly, the ability to form endoderm in response to high activin concentrations, as evidenced by expression of *endodermin* (*a2m*) and *sox17b*, was lost following IBET treatment [*endodermin* (IBET: 100%, *n*=30; DMSO: 0%, *n*=31), *sox17b* (IBET: 100%, *n*=30; DMSO: 0%, *n*=31)] (Fig. 3B). Blastula explants adopt a neural progenitor state when BMP signaling is blocked, for example in response to Chrd, and express genes such as *sox11* and *nrp1*. This transition was also blocked when BET activity was inhibited [*nrp1* (IBET: 100%, *n*=41; DMSO: 3%, *n*=34), *sox11* (IBET: 100%, *n*=35; DMSO: 5.7%, *n*=35)] (Fig. 3C; Fig. S3). Together, these findings are consistent with a model where BET activity is required for the establishment of blastula-stage pluripotency.

### BRD4 is required for neural crest and lineage restriction

Having shown that BET activity is required for pluripotency at blastula stages, we next sought to determine which BET member is essential for this process. Comparison of their expression during early embryogenesis showed that *brd2*, *brd3* and *brd4* have highly similar patterns of expression after blastula stages; however, *brd2* is not expressed before the maternal-to-zygotic transition (MZT) (Fig. S4B). All three BET mRNAs are expressed in the neural plate and neural crest at neurula stages, as well as in migrating and post-migratory neural crest cells. Unique among the BET proteins, BRD4 possesses a C-terminal extension known to interact directly with the P-TEFb complex to promote active transcription (Fig. S4A) (Decker et al., 2017; Palermo et al., 2011). BRD4 is also thought to be the only member of this family with intrinsic enzymatic activity, namely kinase and HAT activity, allowing it to dynamically regulate its interacting partners (Kotekar et al., 2022).

Given its unique attributes, we asked whether loss of BRD4 would phenocopy the effects of IBET treatment. We designed a morpholino that specifically depletes both the s and l alleles of Brd4, but not Brd2 or Brd3 (Fig. S5A,B,F). We found that morpholino-mediated depletion of BRD4 resulted in a loss of neural crest markers (*snai2*: 85%, *n*=86; *foxd3*: 91%, *n*=97) (Fig. S5C,E). BRD4 depletion also led to a failure of blastula explants to undergo proper lineage restriction, as evidenced by increased expression of *sox3* and decreased expression of *krt12.4* at stage 13 [*sox3* (BRD4 MO: 94%, *n*=36; IBET: 100%, *n*=36; DMSO: 0%, *n*=36), *krt12.4* (BRD4 MO: 88%, *n*=41; IBET: 100%, *n*=40; DMSO: 0%, *n*=31)] (Fig. S5D). Co-injection of full length BRD4 mRNA led to a recovery of neural crest gene expression in BRD4 MO neurula-stage embryos (*snai2*: *n*=74; *foxd3*: *n*=68) (Fig. S5C,E). Together these findings suggest that BRD4 is the primary target of IBET and other BET inhibitors in these cells. This is consistent with previous findings that IBET preferentially targets BRD4 over BRD2 and BRD3, and that lymphoma Raji cells stably expressing a dominant negative BRD4 displayed near identical global effects on gene expression to those treated with the BET inhibitor JQ1 (Decker et al., 2017; Filippakopoulos et al., 2010).

### BET activity is required for a subset of gene activation at the maternal-to-zygotic transition

**Fig. 4.**
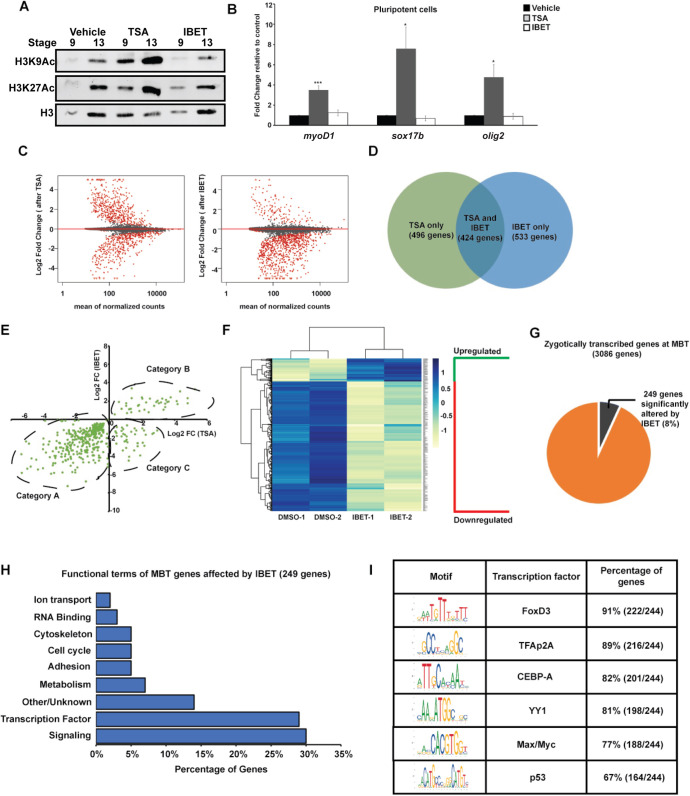
**BET activity is required for activation of a subset of genes at the maternal-to-zygotic transition.** (A) Western blotting for H3K9Ac and H3K27Ac with animal cap explants treated with vehicle, TSA (500 nM) or IBET (250 μM). (B) qRT-PCR for lineage markers with animal cap explants treated with vehicle, TSA (500 nM) or IBET (250 μM). *P<0.05, ***P<0.001 (unpaired, two-tailed t-test). Data are mean±s.e.m. (C) MA-plot depicting global log2 fold gene expression changes in animal cap explants after TSA (left) or IBET (right) treatment. (D) Venn diagram comparing gene sets obtained from RNA-seq of animal cap explants treated with TSA/IBET. (E) Quadrant plot comparing log2 fold changes of genes altered by TSA and IBET. Category A includes genes downregulated by both TSA and IBET, category B includes genes upregulated by both TSA and IBET and category C includes genes upregulated by TSA but downregulated by IBET. (F) Heatmap of genes changed only by IBET but not TSA. (G) Pie chart depicting the genes zygotically transcribed at MZT that are affected by IBET. (H) Functional terms for zygotically transcribed mid-blastula transition (MBT) genes affected by IBET show enrichment for signaling molecules and transcription factors. (I) Motif analysis of promoters of genes altered by IBET at MBT. Enrichment is observed for transcription factor binding of known factors that have been previously characterized to be involved with BET proteins and have known functions in stem cell maintenance and neural crest formation.

Collectively, the functional effects of BET inhibition on blastula and neural crest stem cells appeared to be indistinguishable from the effects of HDAC inhibition (Rao and LaBonne, 2018). This was unexpected given that one treatment increases histone acetylation whereas the other prevents histone acetylation marks from being read. To better understand this, we first asked whether loss of BET activity might indirectly lead to an increase in histone acetylation. As previously reported, treatment of blastula explants with trichostatin A (TSA) leads to an increase in both H3K9Ac and H3K27Ac (Rao and LaBonne, 2018). By contrast, IBET treatment does not alter histone acetylation levels in this assay (Fig. 4A). As our previous work also revealed that HDAC inhibition resulted in aberrant expression of markers of multiple lineage states, and that HDACs control pluripotency by preventing expression of these lineage markers (Rao and LaBonne, 2018), we next asked whether BET proteins regulate pluripotency through a similar mechanism. TSA treatment of blastula explants led to strong increases in *olig2*, *sox17b* and *myoD1* expression; however, IBET had no effect on the expression of these lineage markers (Fig. 4B). This suggested that HDACs and BET proteins may regulate pluripotency through distinct mechanisms.

In order to elucidate how BET proteins impact pluripotency, we next compared the transcriptome changes that occur in response to TSA and IBET treatment. Both TSA and IBET treatment caused global changes in gene expression at blastula stages, with 920 genes differentially expressed in response to TSA treatment as compared with vehicle treatment and 957 genes differentially expressed in response to IBET as compared with vehicle treatment.

Strikingly, although roughly equal numbers of genes were up- versus downregulated in response to TSA treatment, IBET predominantly led to downregulation of gene expression (87.5%) (Fig. 4C). These two inhibitors displayed both distinct and overlapping effects, with 424 genes significantly altered in response to both TSA and IBET treatment (Fig. 4D). These shared targets were binned into three categories: genes downregulated by both TSA and IBET (Category A), genes upregulated by both TSA and IBET (Category B), and genes upregulated by TSA but downregulated by IBET (Category C) (Fig. 4E). Notably, a number of pluripotency genes, including *sall4*, *klf2*, *prdm1*, *prdm14*, *fgfr4*, *lin28a*, *pou5f3*, *sox2*, *sox3* and *tfap2a* fall in category A, whereas a large number of lineage markers, including *plk2*, *not*, *sox17b.2*, *lmo4.2*, *foxi1*, *aplnr* and *cxcr4* fall in category C (Table S1).

Furthermore, of the 957 genes with an altered expression in response to IBET treatment, 533 are unaffected by TSA treatment, and most (85%) of these are downregulated by BET inhibition (Fig. 4F). Together these data provide further evidence that BET proteins regulate the pluripotency of blastula cells through mechanisms distinct to those of HDACs.

It has been suggested that BET activity might play a global role in regulating gene expression during zygotic genome activation (Chan et al., 2019). To examine this possibility in our system, we used published datasets to generate a list of 3086 genes that are not present maternally but become zygotically transcribed at the blastula stages (Johnson et al., 2022; Session et al., 2016). We found that the expression of only 249 of these genes (8%) was significantly altered by IBET treatment (Fig. 4G). This indicates that BET proteins do not globally control zygotic gene activation in *Xenopus*, but instead regulate a key subset of these genes. Interestingly, that subset of genes is enriched for transcription factors and signaling molecules, and motif analysis revealed binding sites for transcription factors associated with the regulation of pluripotency in stem cells, including Foxd3, TFAp2A, CEBP-A, YY1, Max/Myc and p53 (Fig. 4H,I) (Ayaz et al., 2022; Chappell et al., 2013; Di Stefano et al., 2014; Pastor et al., 2018; Respuela et al., 2016; Wang et al., 2018). It is notable that at least three of these factors are shared regulators of both pluripotent blastula and neural crest cells.

### Transcriptome changes in response to BET inhibition are shared across state transitions

**Fig. 5.**
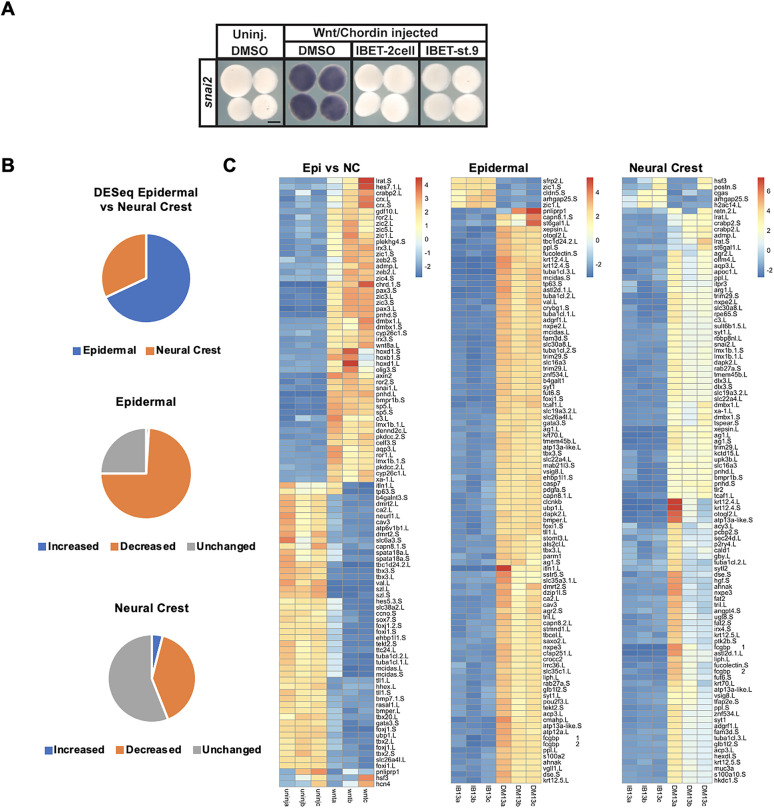
**Epidermal and neural crest progenitor cells possess unique gene signatures.** (A) *In situ* hybridization examining the expression of *snai2* in stage 13 Wnt/Chrd-induced explants treated with vehicle or IBET (250 μM), beginning at the two-cell stage or at stage 9. (B) Pie chart of stage 13 RNA-seq data depicting the percentage of epidermal and neural crest genes determined by comparing uninjected (epidermal) and Wnt/Chrd-induced (neural crest) explants using DESeq2 analysis, and pie charts depicting the effects of IBET treatment on those two different cell populations. (C) Heatmaps of the top 100 genes contributing to the variance between the cell populations compared in the pie charts in B. Scale bars: 250 μm.

To gain further insights into the role of BET activity in establishing the neural crest progenitor state, we examined the transcriptome changes that occur when reprogrammed explants are treated with IBET. First, we confirmed that IBET blocked reprogramming to a neural crest state when treatment was carried out on blastula-stage explants rather than at the two-cell stage, as evidenced by loss of *snai2* expression [IBET (two-cell): 100%, *n*=35; IBET (st. 9): 100%, *n*=29; DMSO: 1.5%, *n*=32] (Fig. 5A). As it did, this allowed us to bypass the requirement for BET activity in establishing blastula-stage pluripotency and focus on its role during lineage decisions. Accordingly, control or neural crest-reprogrammed explants were treated with vehicle or IBET at stage 9 and cultured to neurula stages, when RNA was isolated and used to generate Illumina libraries for transcriptome analysis.

DESeq2 revealed that expression of 2738 genes distinguish the neural crest from the epidermal state at stage 13, 32% of which show higher expression in the neural crest and 68% of which are higher in epidermal progenitors (Fig. 5B). As expected, among the genes most upregulated in the neural crest were known early regulators *sp5*, *pax3*, *zic3*, *foxd3*, and *snai1* (all show log2 fold change ≥8.64 and *P*adj:≤ 2.28 E-07) (Fig. 5C). We next asked which of the genes that distinguish neural crest progenitors from epidermis also showed altered expression in response to IBET treatment. Of the 872 genes that are upregulated in the neural crest at stage 13 relative to epidermis, 349 of them (40%) are downregulated by IBET treatment, further explaining the loss of reprogramming when BET activity is inhibited (Fig. 5B). Of the top 100 annotated genes upregulated in neural crest-reprogrammed caps (compared with controls), the genes for which expression did not decrease in response to IBET tended to be associated with neural development (Fig. S6). Interestingly, 34 of the genes that define the neural crest state at stage 13 are upregulated in response to IBET and among these are the neuronal factors *sox11* and *nestin* as well the Wnt inhibitors *dkk1* and *sfrp2* and the FGF receptor antagonist *spry2*, none of which is functionally associated with a neural crest stem cell state. In addition, inhibition of Wnt/β-catenin signaling has been shown to be a requirement for neural induction (Heeg-Truesdell and LaBonne, 2006).

**Fig. 6.**
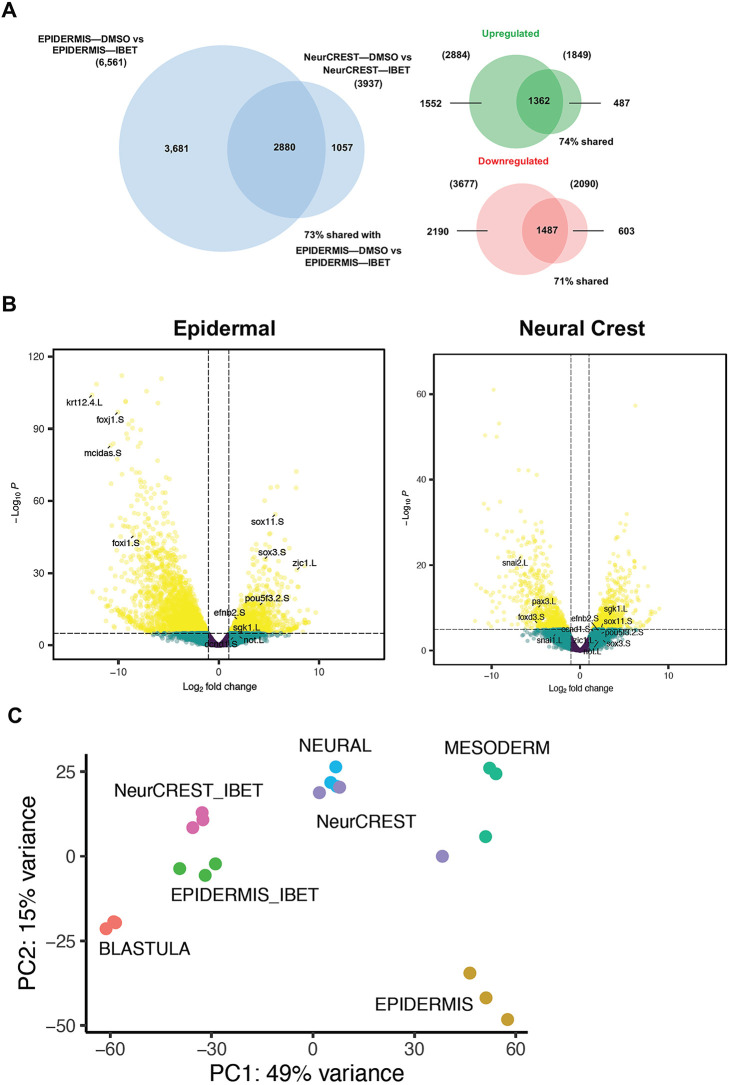
**Transcriptome changes in response to BET inhibition are shared across state transitions.** (A) Venn diagrams depicting the overlap between genes found to be significantly altered using DESeq2 when comparing stage 13 epidermal explant response to IBET (250 μM) with stage 13 neural crest-reprogrammed explant response to IBET. Larger diagram compares total genes sets; smaller diagrams compare upregulated or downregulated gene sets. (B) Volcano plots of genes differentially expressed in stage 13 epidermal and neural crest explants in response to IBET. Genes noted are those that are downregulated in epidermal cells only (*krt12.4.L*, *foxj1.S*, *mcidas.S*, *foxi1.S*), downregulated in neural crest only (*snai2.L*, *snai1.L*, *foxd3.S*, *pax3.L*), and genes which are upregulated in both sets (*efnb2.S*, *ccnd1.S*, *sgk1.L*, *sox11.S*, *sox3.S*, *zic1.L*, *pou5f3.2.S*, *not.L*). (C) PCA plot comparing the variance between the transcriptomes of IBET-treated explants (EPIDERMIS_IBET, NeurCREST_IBET) and that of blastula and several other lineages (Neural, Mesoderm, Epidermis, NeurCREST). Aside from blastula-stage explants, explants of all other lineages were collected at stage 13.

We also examined the full transcriptome changes that occur in response to IBET treatment in cells transiting from pluripotency to either a neural crest or epidermal state. We found that, in response to BET inhibition, 6561 genes are differentially expressed in cells that would otherwise transit to the epidermal state. Significantly fewer genes, 3937, are altered in neural crest reprogrammed explants treated with IBET, and this difference likely reflects the less restricted state of neural crest cells relative to epidermal progenitors. Interestingly, ∼75% of genes for which expression was significantly altered by IBET in the neural crest were also altered in epidermal explants, and their expression was largely changed in the same direction (e.g. upregulated/downregulated) (Fig. 6A). This indicates that many of the genes regulated by BET activity are shared regardless of the state transition.

Perhaps unsurprisingly, the genes downregulated in response to IBET treatment in explants that would otherwise form epidermis were mainly linked to epidermal differentiation including *krt12.4*, *mcidas*, *foxi1* and *foxj1* (Fig. 6B). By contrast, genes upregulated in these explants included genes associated with neural progenitors and pluripotency including *zic1*, *zic2*, *sox11*, *sox3*, *pou5f3.2* and *lhx3*. Gene Ontology analysis of genes upregulated in response to IBET showed enrichment for neural differentiation and, consistent with this, a number of genes associated with neural progenitors were also upregulated in both epidermal and neural crest explants in response to IBET including *sox11*, *sgk1*, *efnb2*, *zic1*, *not* and *ccnd2* at both stages 13 and17 (Fig. 6B; Fig. S7). The upregulation of neural genes in response to BET inhibition is intriguing given our recent finding that the neural progenitor state lies closest in state space to the pluripotent state relative to other non-neural crest lineages (Johnson et al., 2022), a finding that adds genomic context to the neural default model (Smukler et al., 2006; Vallier et al., 2004; Weinstein and Hemmati-Brivanlou, 1997).

To further examine the relationship of IBET-treated explants to a range of embryonic lineage states, we took advantage of recently published transcriptomes for explants induced to form mesodermal or neural progenitors (Johnson et al., 2022). Principal component analysis (PCA) of our current datasets together with transcriptomes of those states at stage 13 shows IBET-treated ‘epidermal’ and ‘neural crest’ explants clustering closely together and closest to neural and pluripotent blastula cells (Fig. 6C), consistent with a role for BET activity in exit from the pluripotent state.

### Inhibiting BET activity prolongs competency to adopt a neural progenitor state

**Fig. 7.**
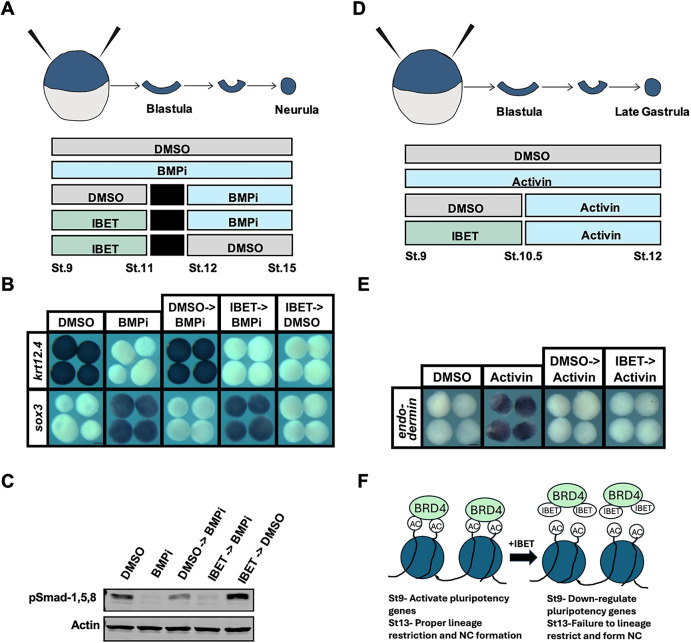
**Inhibition of BET activity prolongs competency to adopt a neural progenitor state.** (A) Diagram of experimental design to test the competence of IBET-treated explants to respond to a neural-inducing BMP inhibitor past the time non-treated explants normally would. (B) *In situ* hybridization examining the expression of *sox3* and *krt12.4* in explanted blastula caps treated with vehicle or IBET (250 μM) from stages 9 to 11, washed into fresh 1× MMR, treated with BMPi (K02288) (20 μM) from stages 12 to 15 and then collected for analysis. Only explants treated with IBET then BMPi were able to induce a neural fate (as evidenced by expression of *sox3* but not *krt12.4*) similar to that of explants treated with just BMPi. (C) Western blot analysis of stage 15 explants collected at same time as explants collected for *in situ* analysis with the same treatment schedule. Blot was probed with anti-pSmad-1,5,8 to assess levels of active BMP signaling, and anti-actin for normalization. pSmad-1,5,8 and actin were detected on the same membrane using the same secondary antibody. (D) Diagram of experimental design to test the competence of IBET-treated explants to respond to endoderm-inducing activin past the time non-treated explants normally would. (E) *In situ* hybridization examining expression of *endodermin* in explanted blastula caps treated with vehicle or IBET from stages 9 to 10.5, then treated with activin from stages 10.5 to 12, then collected for analysis. Only explants treated with activin at stages 9 through 12 showed increased expression of *endodermin*. (F) Model depicting two temporally distinct roles for Brd4 in the establishment and exit from pluripotency. Scale bars: 250 μm.

The above findings indicate that at stage 13, IBET-treated explants share features with both pluripotent blastula cells and neural progenitor cells. This raised the intriguing possibility that blocking BET activity might extend the competence of initially pluripotent cells to transit to a neural progenitor state. Our recent work demonstrated that stage 10.5, early gastrulation, is when cells are initially diverted from a neural trajectory and begin to acquire epidermal character (Johnson et al., 2022). We therefore asked whether IBET treatment of blastula explants would allow them to respond to neural inducing cues past the time when control explants can. Treatment with a small molecule antagonist of BMP signaling, K02288 (Sanvitale et al., 2013), directs blastula explants to adopt a neural fate, as evidenced by expression of *sox3* (K02288: 100%, *n*=43; DMSO: 0%, *n*=34), and prevention of *krt12.4* expression (K02288: 0%, *n*=38; DMSO: 100%, *n*=42) at stage 13 (Fig. 7A,B; Fig. S8). When BMP inhibition is initiated at stage 12, during gastrulation, cells are no longer competent to adopt a neural fate, as evidenced by lack of *sox3* induction (0%, *n*=41) and instead express *krt12.4* (100%, *n*=45). By contrast, explants treated with IBET from stages 9 to 11 were able to adopt a neural fate when BMP signaling was inhibited (BMPi) at stage 12 and, accordingly, these explants expressed *sox3* (100%, *n*=45) and not *krt12.4* (0%, *n*=37). This was accompanied by a restored loss of phosphorylated R-Smad proteins (Fig. 7C). Further, we did not find that IBET and BMPi had similar effects on *sox3* expression or pSmad-1,5,8 levels in explants treated from stages 9 to 15 compared with controls (Fig. S9).

To determine whether IBET-treated explants retain full pluripotency or only a prolonged competence to respond to neural-inducing cues, we asked whether they also exhibited a prolonged ability to respond to activin treatment by forming endoderm. Treatment of blastula explants with activin at stage 9 leads to strong expression of *endodermin* at stage 12 (100%, *n*=39); however, these explants can no longer form endoderm when treated with activin at stage 10.5 (100%, *n*=33) (Fig. 7D,E). Importantly, blocking BET activity had no effect on the ability of activin to induce endoderm at stage 10.5, as evidenced by a failure to induce expression of *endodermin* (100%, *n*=34), nor did it affect the ability of activin to increase pSmad-2 levels (Fig. S9). Thus, the effect of BET inhibition at blastula stages is to prolong the competence of initially pluripotent cells to transit to a neural progenitor state, and not to prolong full pluripotency (e.g. the ability to adopt all lineage states) (Fig. 7F).

## DISCUSSION

Epigenetic regulation is a crucial component of the regulatory mechanisms that control stem cell attributes. Histone post-translational modifications (PTMs), together with the chromatin remodelers tasked with writing, erasing and reading these marks, play essential roles in the formation and maintenance of both pluripotent blastula and neural crest stem cells. We recently reported that a low level of histone acetylation is a shared feature of both neural crest cells and pluripotent blastula cells, and that HDAC activity is crucial for establishment of both cell types (Rao and LaBonne, 2018). That study further found that increasing HDAC activity enhanced reprogramming to a neural crest state. Moreover, Snai2 has been shown to form a complex with HDACs and the adaptor LMO4 to control expression of some neural crest regulatory factors in *Xenopus* (Ochoa et al., 2012). Together those findings pointed to the importance that control of histone acetylation plays in both the neural crest and blastula stem cells and raised the important question of what proteins read these marks to control chromatin accessibility and gene expression.

To address this question, we investigated a class of epigenetic factors containing bromodomains that selectively target acetylated lysines and recruit transcriptional regulatory machinery (Marmorstein and Zhou, 2014). Interestingly, these readers of histone acetylation have also been shown to be crucial for the maintenance of pluripotency in cultured ESCs, providing further evidence that histone acetylation is under tight control during lineage restriction. More specifically, the BET family bromodomain reader, Brd4, has been shown to interact with acetylated H4 to regulate pluripotency in ESCs as well as to interact with Oct4 (Gonzales-Cope et al., 2016; Wu and Belmonte, 2015). Brd4 knockout in mice is embryonically lethal, and it has been shown that Brd4 activity is required to maintain the pluripotent state in ESCs (Di Micco et al., 2014; Fernandez-Alonso et al., 2017; Houzelstein et al., 2002; Liu et al., 2014). Moreover, Brd4 activity has been closely linked to regulation of Myc, which plays a key role in both blastula and neural crest stem cells (Bellmeyer et al., 2003; Kotekar et al., 2022).

In this study, we found that *Xenopus* embryos treated with small molecules that block binding of BET domains to acetylated lysine residues on histones (Filippakopoulos et al., 2010; Mirguet et al., 2013; Rhyasen et al., 2016) showed a severe reduction or complete loss of neural crest markers. Notably, treatment with these inhibitors did not result in a loss of neural progenitor cells in whole embryos (Fig. 1B). Inhibiting BET activity also prevented animal pole explants from being reprogrammed to a neural crest state in response to Wnt and Chrd expression. In addition to defects in neural crest progenitors, we found that BET inhibition led to downregulation of pluripotency factors in blastula stem cells (Fig. 2A) and the loss of functional pluripotency in explanted animal pole cells (Fig. 3), indicating that a requirement for BET activity is another shared feature of blastula and neural crest stem cells. Morpholino-mediated depletion of Brd4 supports that the inhibition of this BET factor contributes most to the effects of IBET and the other inhibitors (Fig. S5).

The findings that BET activity is essential for pluripotency in blastula stems cells and in neural crest cells was puzzling, however, as it meant that blocking the activity of a reader of acetylation appeared to produce the same phenotype at a functional level as the loss of HDAC activity, which increases histone acetylation (Rao and LaBonne, 2018). To characterize the mechanisms through which these two inhibitors regulate pluripotency, we compared the transcriptome changes elicited by TSA and IBET in blastula stem cells. Both inhibitors drove global changes in gene expression, with 957 genes differentially regulated after IBET treatment and 920 genes significantly differentially regulated in blastula cells after TSA treatment. However, although HDAC inhibition led to roughly equal numbers of genes being upregulated versus downregulated, BET inhibition predominantly downregulated gene expression (Fig. 4C), evidence pointing to distinct mechanisms of action. That HDAC inhibition led to roughly equal numbers of genes being up- and downregulated was of interest given that HDACs are thought to predominantly be repressors of gene expression. The dynamic changes in the transcriptome in response to TSA treatment suggests that HDACs may play a more context-specific and complex role in the regulation of gene expression. Indeed, recent work has found that HDACs are enriched at the promoters of active genes (Wang et al., 2009), and that HDACs may play a positive regulatory role in stem cell maintenance, as we find here (Baltus et al., 2009; Saunders et al., 2017).

It was noteworthy that fewer than one third of genes with an altered expression in response to HDAC or BET inhibition were shared (Fig. 4D), and there were 533 genes differentially expressed in response to IBET but not TSA treatment. Among the most significantly downregulated of these genes were *zic3*, *admp*, *dlx3*, *bmp7* and *ventx1.2* (Table S1). Although our findings indicate that IBET treatment is impacting gene expression around the time when zygotic gene expression is first initiated (Fig. 4G), only 8% of genes zygotically transcribed at mid-blastula stages were significantly altered by BET inhibition, indicating that BET activity does not globally control zygotic gene activation but rather acts more selectively. This finding contrasts with recent work in zebrafish that found that 84% of zygotically transcribed genes were reduced in expression more than fourfold following JQ1 treatment (Chan et al., 2019). This difference may be explained by work showing that histone methylation, a histone PTM that is not recognized by BET proteins, also plays a crucial role in zygotic gene activation in *Xenopus* (Blythe et al., 2010). Β-Catenin recruitment of the arginine methyltransferase Prmt2 to target promoters results in dimethylation of histone H3 arginine 8, which is necessary and sufficient to establish the dorsal gene expression program. Genes in this program, such as *siamois* (*sia1*) and *xnr3* (*nodal3.1*), were not found to be altered by IBET, suggesting that BET protein function during zygotic gene activation is specific to the activation of neural/pluripotency gene expression.

Further insights into the role of BET activity in controlling stem cell attributes was gained by examining the effects of BET inhibition on explants reprogrammed to a neural crest progenitor state. We found that 2738 genes were differentially expressed in explants reprogrammed to a neural crest state by Wnt/Chrd compared with non-reprogrammed explants. Of these genes, 872 were upregulated, and we characterize these as the genes that define the neural crest/neural plate border state at stage 13 including *zic1*, *pax3*, *sp5*, *foxD3* and *snai1*. When neural crest-reprogrammed explants were treated with vehicle or IBET at stage 9 and transcriptome changes quantified at neurula stages, 40% of the genes that define the neural crest state were downregulated in response to BET inhibition, including major known regulators *foxd3*, *pax3*, *snai1*, *snai2*, *sox8*, *sox9* and *tfap2a* (Fig. 5B,C).

When we compared the transcriptome changes that occur in response to IBET treatment in cells transiting from pluripotency to either a neural crest or epidermal state, it was striking that significantly fewer genes showed altered expression in the neural crest, potentially reflecting the less differentiated state of these cells relative to epidermal progenitors and a shared gene regulatory network (GRN) architecture with blastula stem cells. Among the epidermal genes most strongly downregulated by IBET were a number of keratins including Krt12.4, the transcription factor Tp63, Mcidas, a cell cycle protein associated with multi-ciliated cells, Xepsin, an epidermis-specific protease, and Foxj1, a key transcriptional regulator of multi-ciliated cells. Somewhat puzzlingly, among the genes most upregulated in prospective epidermis in response to IBET were *hnf1b*, a transcription factor best known for its role in kidney development, *hemicentin 1*, a fibulin family extracellular matrix protein, and *claudin 5*. Also upregulated were neuronal genes including *grin3b*, *nsmf*, *trpv1*, *marchf4*, *nkx2-2*, *ncam2*, genes associated with a neural progenitor state as well as pluripotency such as *zic1*, *zic2*, *zic4*, *zeb2*, *sox11* and *pou5f3.2*, and regulators of key developmental signaling including *sfrp2*, *efna2*, *mob3c*, *ptch2*, *fzd7*.

We used PCA to compare the datasets from this study to recently published transcriptomes for mesodermal and neural progenitors (Johnson et al., 2022). This PCA analysis indicated that IBET-treated epidermal and neural crest explants clustered closely together and closest to neural and pluripotent blastula cells. This was consistent with our finding that at neurula stages the neural plate is expanded in IBET-treated embryos (Fig. 1; Fig. S2), and that epidermal as well as neural crest reprogrammed explants displayed upregulation of genes associated with both pluripotency and the neural progenitors in response to BET inhibition. Our recent work provided evidence that the neural progenitor state lies closest in state space to pluripotent blastula cells compared with other lineages examined, with respect to both gene expression and transcriptome dynamics (Johnson et al., 2022). That work suggested that the onset of gastrulation can be considered a point in time when a group of equipotent cells (neural/epidermal) diverge and either continue changing state to become epidermal cells or do not continue changing state and default to neural cells. The finding that IBET-treated neurula-stage explants share features with both pluripotent blastula cells and neural progenitor cells regardless of lineage (epidermal versus neural crest) raised the possibility that blocking BET activity might extend the competence of initially pluripotent cells to transit to a neural progenitor state. Indeed, we found that by late gastrulation (stage 12) control explants are no longer competent to form neural progenitors in response to BMP inhibition, although if initially pluripotent explants are first treated with IBET (through stage 11) they remain competent to form neural tissue at least through stage 12 (Fig. 7B), a finding which could have implications for regenerative medicine. This is not, however, a complete retention of functional pluripotency, as similarly treated explants do not exhibit prolonged competence to give rise to endoderm in response to activin (Fig. 7E,F). These findings are consistent with studies of mouse ESCs that found when they were grown in the absence of exogenous factors or feeder layers, the majority (82%) of surviving cells expressed the neural marker nestin, suggesting that when pluripotency is lost/blocked, stem cells retain the ability to contribute largely to neural cell populations but not other lineages (Tropepe et al., 2001).

Going forward it will be important to identify the mechanisms through which BRD proteins regulate a specific subset of genes during zygotic genomic activation and compare that to the genes they directly control during the exit from pluripotency. This may be through interaction with specific binding partners, including neural crest/pluripotency factors. Similarly, it will be important to better understand the mechanistic connection between their role in pluripotency and in the neural crest. Data from this study and others suggest that the effects on global gene expression of pan-BET inhibition are likely due specifically to a loss of Brd4, although this prediction has not been tested thoroughly (Decker et al., 2017). Further studies comparing the activities of Brd2, Brd3 and Brd4 individually, particularly those involving domain swaps of the C terminus of Brd4, which is thought to confer differences in functionality between Brd4 and other BET members, onto Brd2 and Brd3 may be revealing as to how these proteins regulate pluripotency. Such experiments are complicated, however, by the large size of Brd4 (200 kDa), which makes expressing full-length protein to compare the functions of BET mutants challenging (Drumond-Bock et al., 2022). BET family proteins are of significant clinical relevance and BET inhibitors have shown promise in targeting neuroblastoma, a neural crest-derived cancer that is the most common pediatric solid tumor (Delmore et al., 2011). Moreover, dysregulation of histone acetylation has been linked to neural crest-related congenital defects including cleft lip and palate, and cardiovascular defects (Alsdorf and Wyszynski, 2005; Wyszynski et al., 2005). The current study provides novel insights into the functional roles of a key reader of histone acetylation marks in the maintenance of pluripotency and the establishment of neural crest stem cells and the transcriptome datasets generated may shed further light on these and other neurocristopathies.

## MATERIALS AND METHODS

### Embryological methods

Wild-type *Xenopus laevis* embryos were staged and collected in accordance with standard methods (Nieuwkoop et al., 2020). *In situ* hybridizations were performed on embryos and explanted animal caps using previously described methods (LaBonne and Bronner-Fraser, 1998). Microinjection of mRNA (Ambion, mMessage mMachine SP6 Transcription Kit) or morpholino (Gene Tools) was carried out in 1-4 cells at the two- to eight-cell stage as previously described (Lee et al., 2012) and manipulated embryos were then cultured in 0.1× Marc's Modified Ringer's Solution (MMR) [0.1 M NaCl, 2 mM KCl, 1 mM MgSO_4_, 2 mM CaCl_2_, 5 mM HEPES (pH 7.8), 0.1 mM EDTA] until being collected or dissected for animal cap explant assays. All animal cap explants were manually dissected during the early blastula stage and then cultured in 1× MMR until collection. For activin experiments, animal cap explants were dissected and immediately cultured in 1× MMR with 0.1% bovine serum albumin (BSA) and recombinant activin protein (R&D Systems) at a final concentration of 20-40 ng/ml for mesoderm induction and 100 ng/ml for endoderm induction. Manipulated embryos and/or explants were fixed in 1× MEM [100 mM MOPS (pH 7.4), 2 mM EDTA, 1 mM MgSO_4_] with 4% formaldehyde and dehydrated in methanol before *in situ* hybridizations. Results shown are representative of a minimum of three biological replicates.

### DNA constructs and inhibitor treatments

Full length *Xenopus brd2*, *brd3* and *brd4* clones were obtained from the *Xenopus* ORFeome (www.xenbase.org/reagents/static/orfeome.jsp) and subcloned into pGEM-T vector for synthesis of RNA probes. Full length *brd2, brd3* and *brd4*, and the short isoform of brd4, were subcloned into pCS2 vector for synthesis of mRNA for microinjection. For BET protein activity inhibition, embryos and/or animal cap explants were treated with IBET762, JQ1 or AZD5153 (Sigma-Aldrich) at final concentrations of 250 µM, 10 µM and 100 µM, respectively, in 0.1× or 1× MMR solution. For HDAC protein activity inhibition, embryos and/or animal cap explants were treated with TSA at a final concentration of 500 nM and BMP signaling activity was inhibited with K02288 at a final concentration of 20 µM.

### Western blot analysis

Five whole embryos or 10-12 animal explants per condition were lysed in TNE lysis buffer [50 mM Tris-HCl (pH 7.4), 150 mM NaCl, 0.5 mM EDTA and 0.5% Triton X-100] supplemented with protease inhibitors [Aprotinin, Leupeptin and phenylmethylsulfonyl fluoride (PMSF)] and a complete Mini tablet (Roche). Proteins were resolved by SDS-PAGE, blotted and then probed for using the following antibodies: anti-H3K9Ac (#9649, Cell Signaling Technology, 1:2000), anti-H3K27Ac (ab4729, Abcam, 1:2000), anti-H3 (#3638 and #4499, Cell Signaling Technology, 1:1000), anti-Myc (9E10, Santa Cruz Biotechnology, 1:3000), anti-phospho-Rsmad-1/5/8 (Ser463/465, Sigma-Aldrich, 1:1000), anti-phospho-Smad2 (Ser465/467, Sigma-Aldrich, 1:500) and anti-actin (A2066, Sigma-Aldrich, 1:5000). IRDye secondary antibodies (LI-COR, 926-32210/926-68021, 1:20,000) were then used to detect proteins using the Odyssey platform (LI-COR Biosciences). Protein amounts were quantified using the Image Studio Lite software (LI-COR Biosciences), and normalized to H3 (H3K9AC and H3K27Ac) or actin (Myc, phospho-Rsmad-1/5/8 and phospho-Smad2).

### RNA isolation, cDNA synthesis and qRT-PCR

RNA isolation, cDNA synthesis and qRT-PCR were performed as previously described (Buitrago-Delgado et al., 2015). The primers used for the *ODC1*, *sox3*, *krt12.4* and *nrp1* genes have been described previously (Geary and LaBonne, 2018). *sox3*, *krt12.4* and *nrp1* expression were normalized to *ODC1* and the fold change was calculated relative to control samples collected at the same stage. Results show the mean average of three biological replicates and error bars depict the standard error of the mean. An unpaired, two-tailed *t*-test was used to determine statistical significance.

### Bioinformatic analyses

The obtained reads were checked for quality using FAST-QC (Babraham Bioinformatics) and aligned to the *Xenopus laevis* genome 9.2 (Xenbase) using STAR aligner (Dobin et al., 2013). The aligned reads were counted using HTSeq Counts (Anders et al., 2015).

Differential analysis was carried out in R using the DESeq2 package applying standard log-fold shrinkage procedures and TSA dataset and all IBET datasets were processed separately with their own controls (Love et al., 2014). Genes were considered significantly changed when *P*adj <0.05. For the zygotic gene activation analysis, egg-stage, stage 8 and stage 9 data were obtained from Xenbase (Session et al., 2016). The IBET and zygotic gene activation datasets were matched to find the overlapping genes, and only those were considered for the analysis. The zygotic gene activation dataset was filtered to remove genes which did not have transcripts per million (TPM) >0.5 in any of the three stages. Genes zygotically transcribed were identified based on the criteria of stage8 TPM/egg stage or stage9 TPM/egg stage >5.

### Animals

All animal procedures were approved by the Institutional Animal Care and Use Committee, Northwestern University, and are in accordance with the National Institutes of Health Guide for the Care and Use of Laboratory Animals.

## Supplementary Material

10.1242/develop.202990_sup1 Supplementary information

Table S1. Genes significantly changed by TSA and IBET List of genes significantly altered in response to both TSA and IBET by category
