# Supplementary material for: BET activity plays an essential role in control of stem cell attributes in Xenopus
Source: Development. 2024 Jul 3;151(13):dev202990. doi: 10.1242/dev.202990 (PMC11266789; doi:10.1242/dev.202990)
Supplement: Supplementary information [file develop-151-202990-s1.pdf]

Supplemental Figure 1

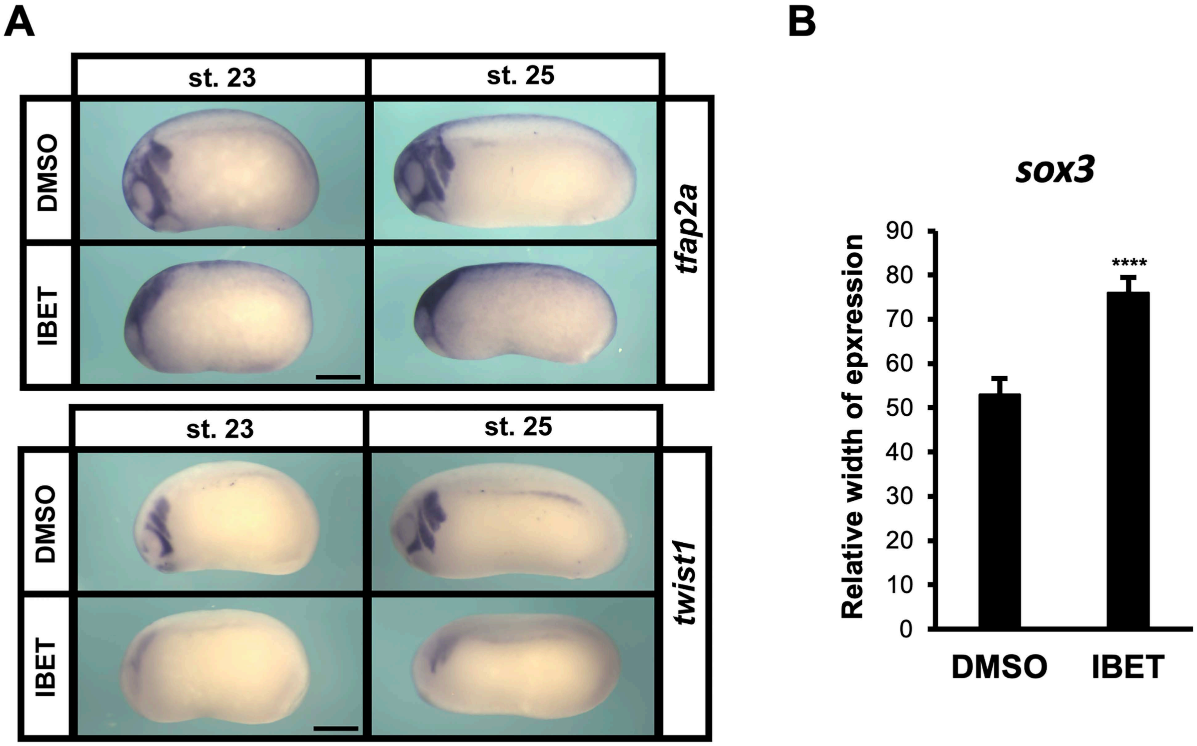

Fig. S1. A) *In situ* hybridization examining the expression of neural crest factors *tfap2a* and *twist* following treatment with vehicle or IBET [250 μM]. All embryos were treated at the two-cell stage and were collected at stages 23 or 25. After stage 23 IBET treated embryos were consistently smaller than controls. **(B)** Graph depicting width of *sox3* expression (measured at point of greatest width of *sox3* expression) relative to whole embryo width at same region in embryos from figure 1b. For determining statistical significance, a standard two-tailed T-test with two sample equal variance was used. Asterisks denote significant p values (*p ≤ 0.05, **p ≤ 0.01, ***p ≤ 0.001, ****p ≤ 0.0001). Scale bars: 500 μm.

Supplemental Figure 2

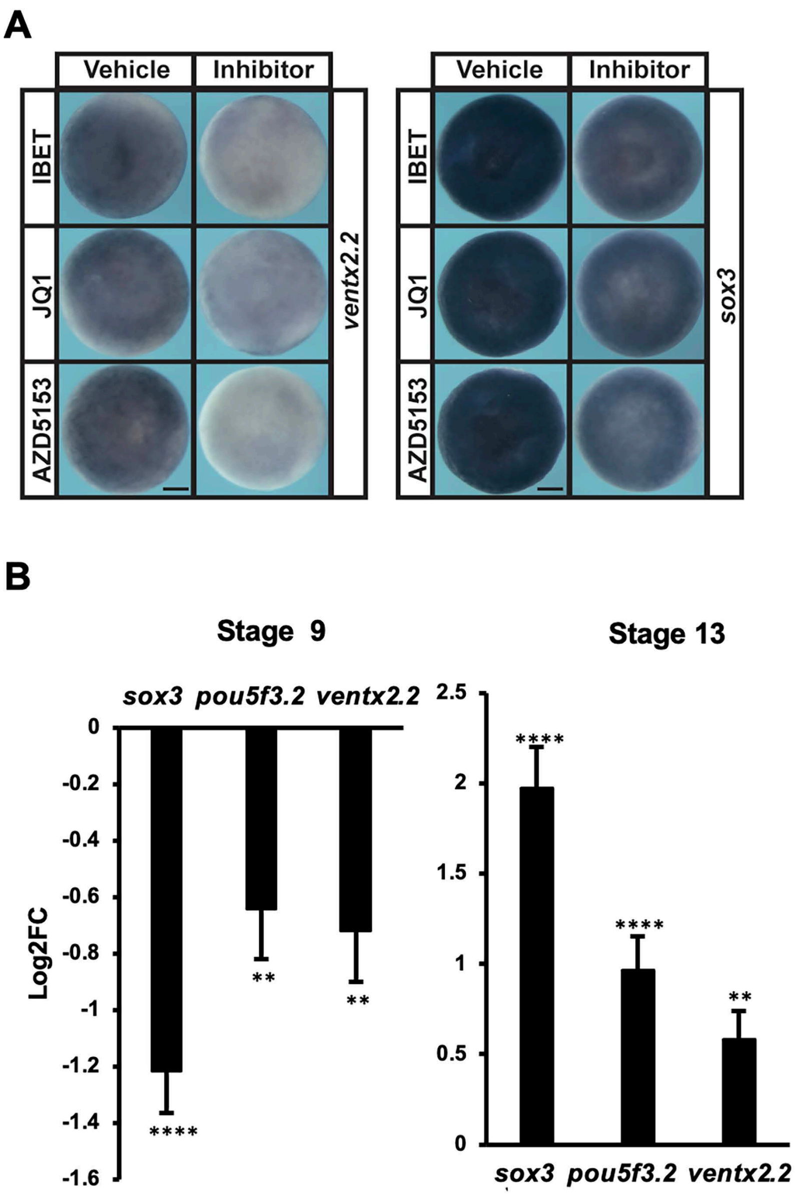

Fig. S2. (A) *In situ* hybridization examining the expression of *ventx2.2* or *sox3* in pluripotent blastula cells following treatment with vehicle or inhibitor [250 μM IBET, 10 μM JQ1, 100 μM AZD5153]. Embryos were treated at the two-cell stage and collected at late blastula stages (stage 9). **(B)** Graphs depicting DESeq results of IBET-treated [250 μM] explants relative to control explants (Log2FC) for pluripotency genes *sox3*, *pou5f3.2*, *ventx2.2* at stages 9 and 13. Error bars represent the standard error of log fold change. Explants were treated at the two-cell stage, then grown in culture with sibling embryos and collected at late blastula stages (stage 9) and early neurula stages (stage 13) for RNA isolation. Scale bars: 250 μm.

Supplemental Figure 3

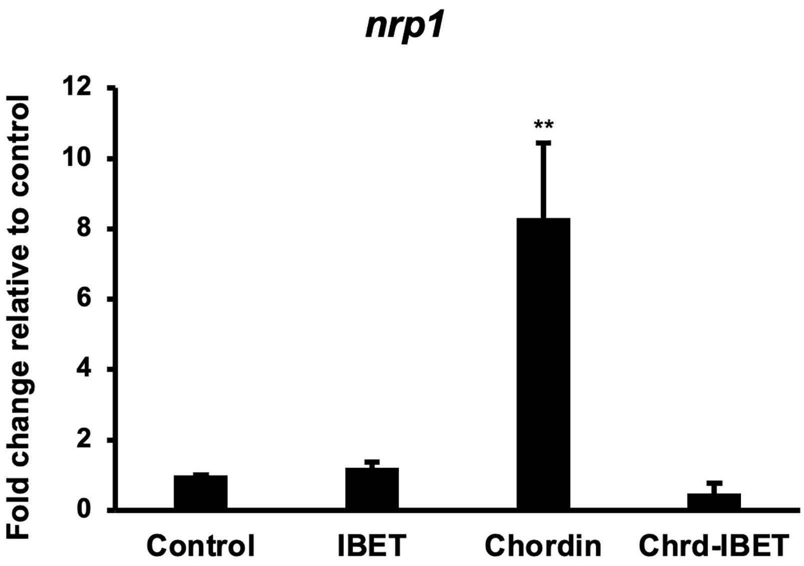

Fig. S3. Graph depicting results from qRT-PCR of explanted blastula caps examining the expression of *nrp1* following treatment with vehicle or IBET [250 μM], with or without microinjection of chordin mRNA. Explants were injected with chordin and treated at the two-cell stage, then explants were grown in culture with sibling embryos and collected at late neurula stages (stage 18). For determining statistical significance, a standard two-tailed T-test with two sample equal variance was used. Asterisks denote significant p values (*p ≤ 0.05, **p ≤ 0.01, ***p ≤ 0.001, ****p ≤ 0.0001).

Supplemental Figure 4

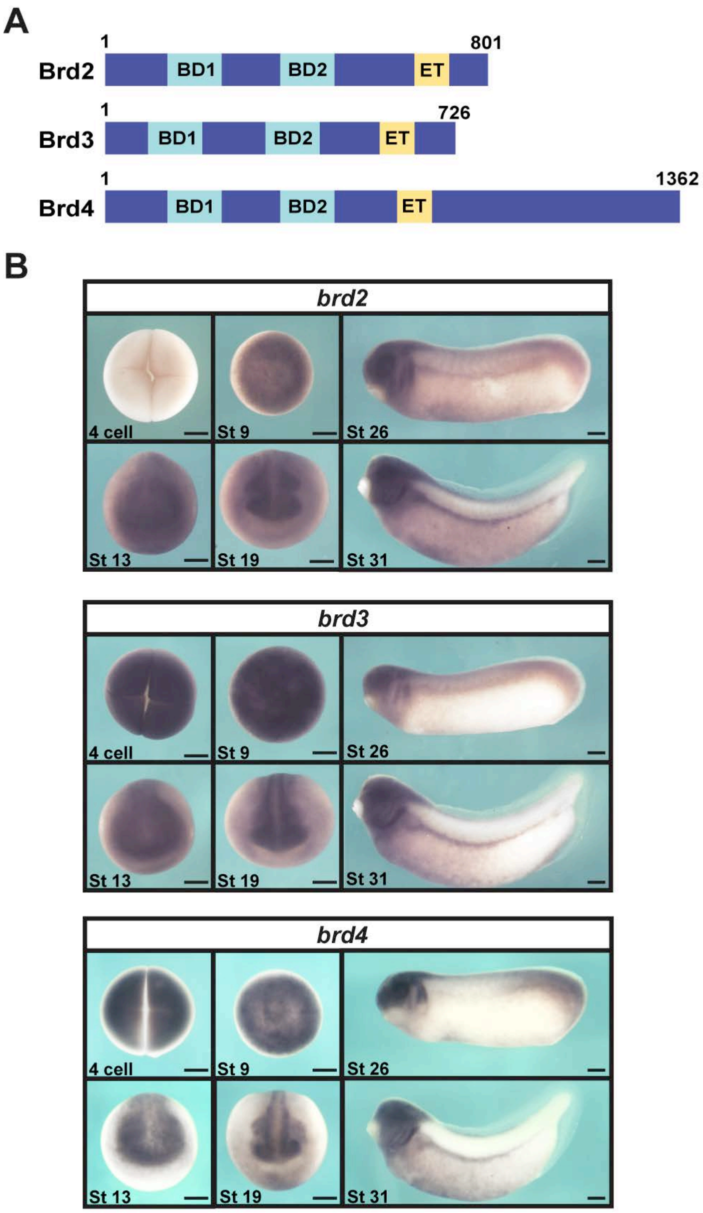

Fig. S4. (A) Schematic of BET family member Brd2, Brd3,and Brd4 proteins with position of bromodomains (BD1 and BD2) and extra terminal (ET) domains indicated. **(B)** *In situ* hybridization examining the expression of brd2, brd3, and *brd4* in early wild type embryos collected at the four-cell stage and stages 9, 13, 19, 26, and 31. Scale bars: 250 μm.

Development • Supplementary information

Supplemental Figure 5

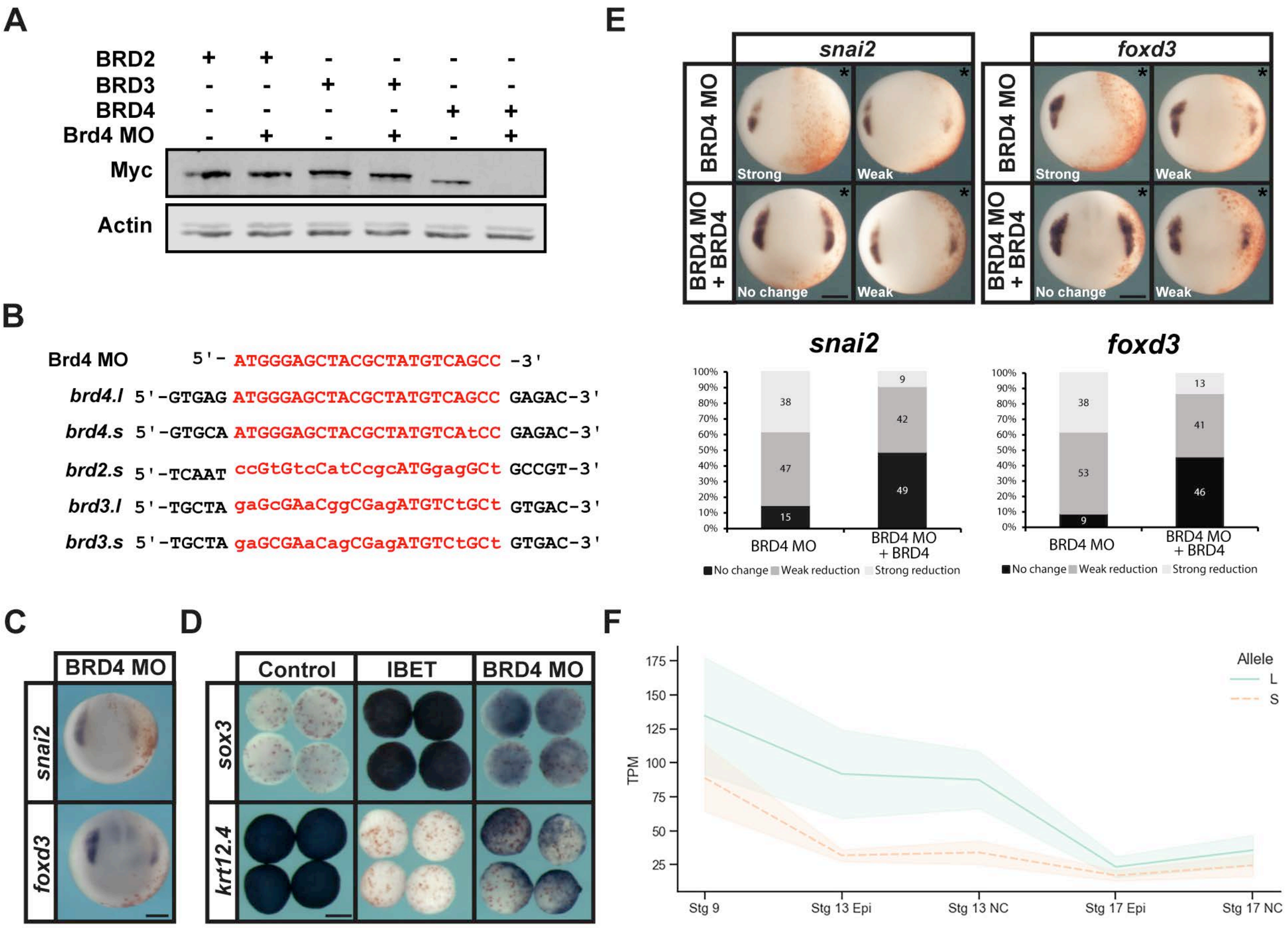

Fig. S5. (A) Western blot analysis probing for myc or actin in blastula explants from embryos injected with mRNAs for myc-tagged *brd2*, *brd3*, or *brd4* (short isoform), with or without morpholino targeting BRD4. **(B)** Alignment of BET family gene transcription start sites with BRD4 MO binding site. Red letters denote exactly where sites align, capital letters are bases which directly match the MO site binding site, and lower-case letters denote a mismatch.
(C) *In situ* hybridization examining the expression of neural crest factors *snai2* and *foxd3* in embryos after morpholino-mediated depletion of BRD4. **(D)** *In situ* hybridization examining the expression of *sox3* and *krt12.4* in explanted blastula caps treated with vehicle or IBET [250 μM], or depleted of BRD4 by morpholino. Embryos were treated at the two-cell stage, then explants were grown in culture with sibling embryos for staging purposes and collected at early neurula stages (stage 13). **(E)** *In situ* hybridization examining the expression of neural crest factors *snai2* and *foxd3* in embryos after morpholino-mediated depletion of Brd4, and rescued by co-injection with *brd4* mRNA. Graph depicts the percentage of embryos displaying the indicated affect (no change, weak reduction, or strong reduction) on the expression of *snai2* and *foxd3* after morpholino-mediated depletion of Brd4, and rescued by co-injection with *brd4* mRNA. **(F)** Graph depicting average TPMs of *brd4.l* and *brd4.s* alleles in stage 9 blastula, stage 13 epidermis and neural crest, and stage 17 epidermis and neural crest explants. Error bars represent the standard error of mean. Scale bars: 250 μm.

Development • Supplementary information

Supplemental Figure 6

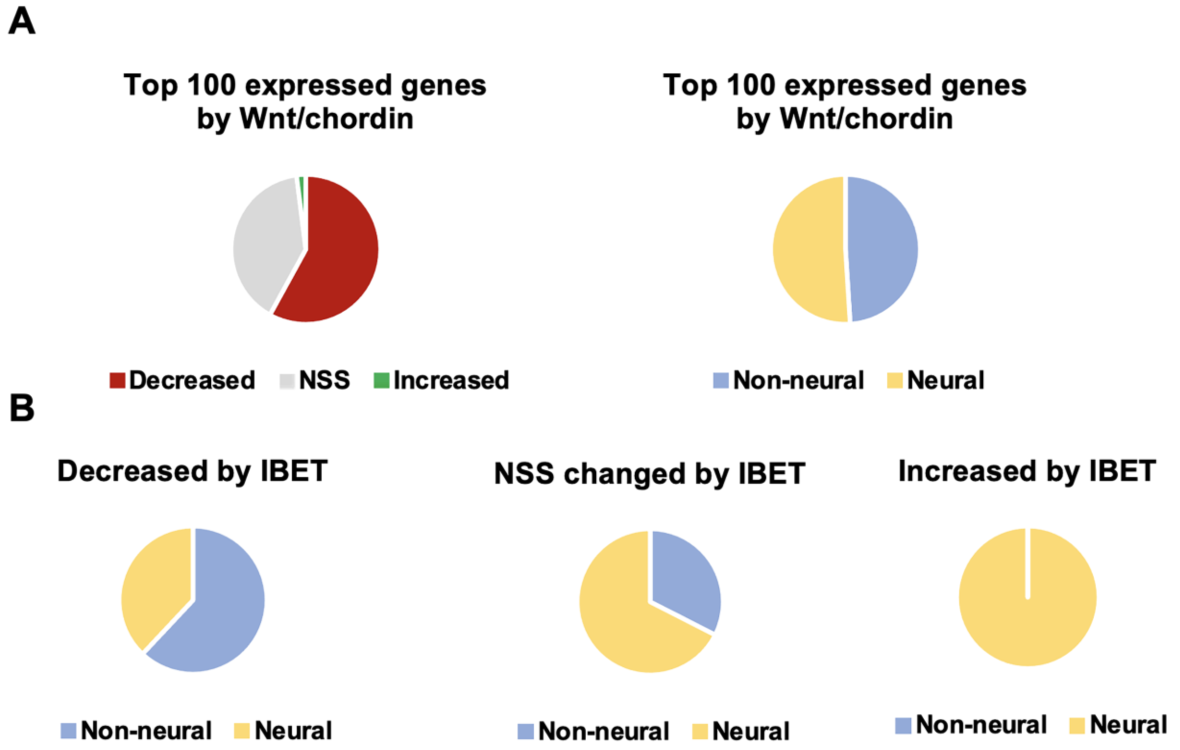

Fig. S6. (A) Pie charts depicting percentages of the top 100 highly expressed genes in stage 13 neural crest-reprogrammed explants (determined by DESeq analysis comparing non-treated epidermal explants to non-treated neural crest-reprogrammed explants) which are decreased, not statistically significantly altered by (NSS), or increased by IBET (left), and which are classified as non-neural or neural genes (right). (B) Pie charts depicting the percentage of non-neural and neural genes amongst those 100 highly expressed neural crest genes decreased by IBET (left), NSS changed by IBET (center), and increased by IBET (right).

Development • Supplementary information

Supplemental Figure 7

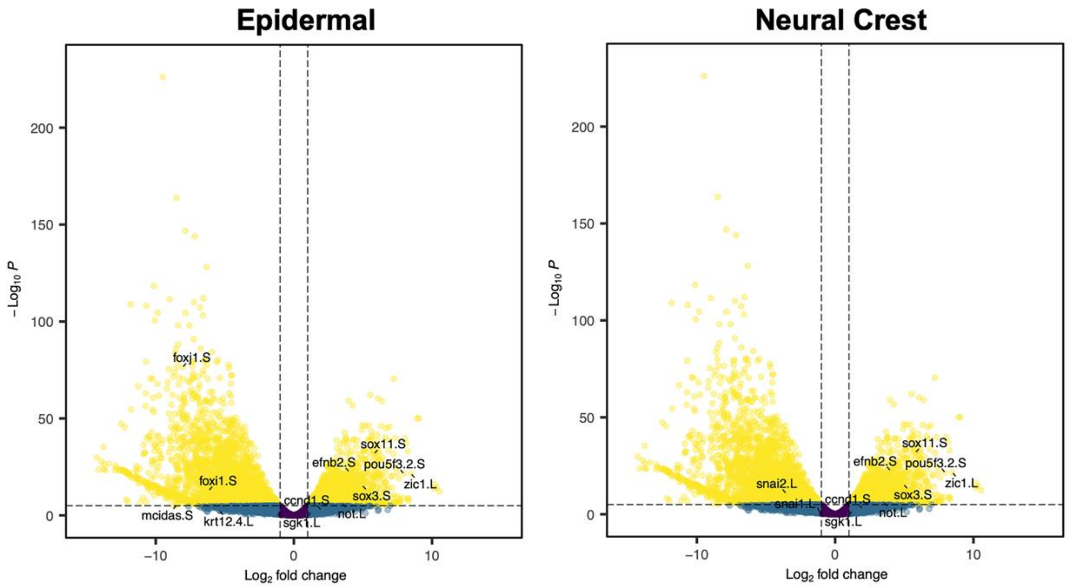

Fig. S7. Volcano plots of genes differentially expressed in stage 17 epidermal (left) and neural crest (right) explants in response to IBET. Genes noted are those that are downregulated in epidermal only (*krt12.4.L, foxj1.S, mcidas.S, foxi1.S*), downregulated in neural crest only (*snai2.L, snai1.L*), and genes which are upregulated in both sets (*efnb2.S, ccnd1.S, sgk1.L, sox11.S, sox3.S, zic1.L, pou5f3.2.S, not.L*).

Supplemental Figure 8

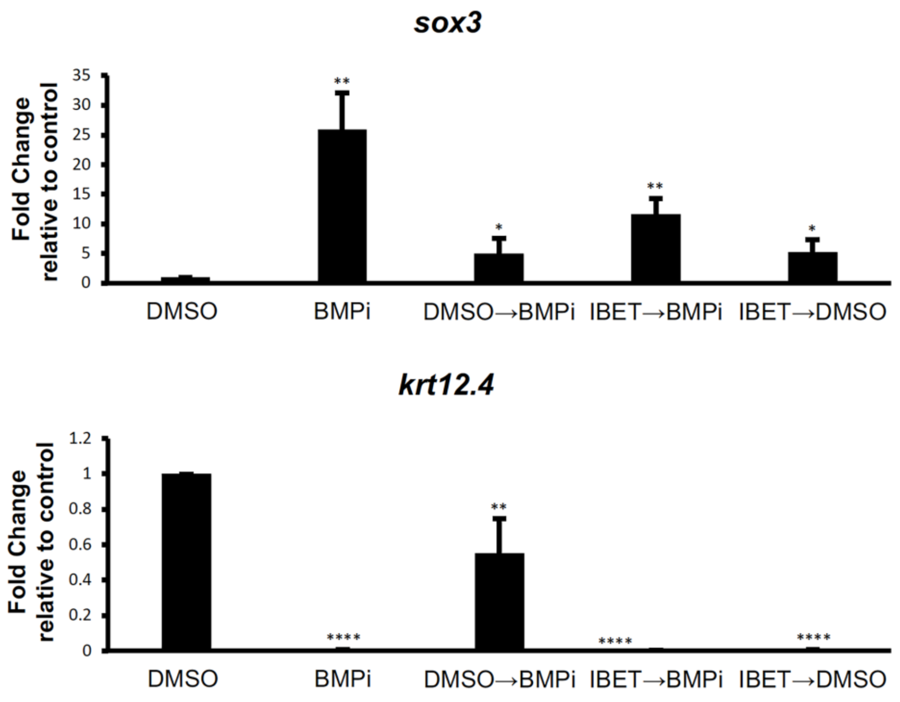

Fig. S8. Graphs depicting qRT-PCR results of explanted caps treated identically as those in figure 7b. For determining statistical significance, a standard two-tailed T-test with two sample equal variance was used. Asterisks denote significant p values (*$p \leq 0.05$, **$p \leq 0.01$, ***$p \leq 0.001$, ****$p \leq 0.0001$).

Development • Supplementary information

Supplemental Figure 9

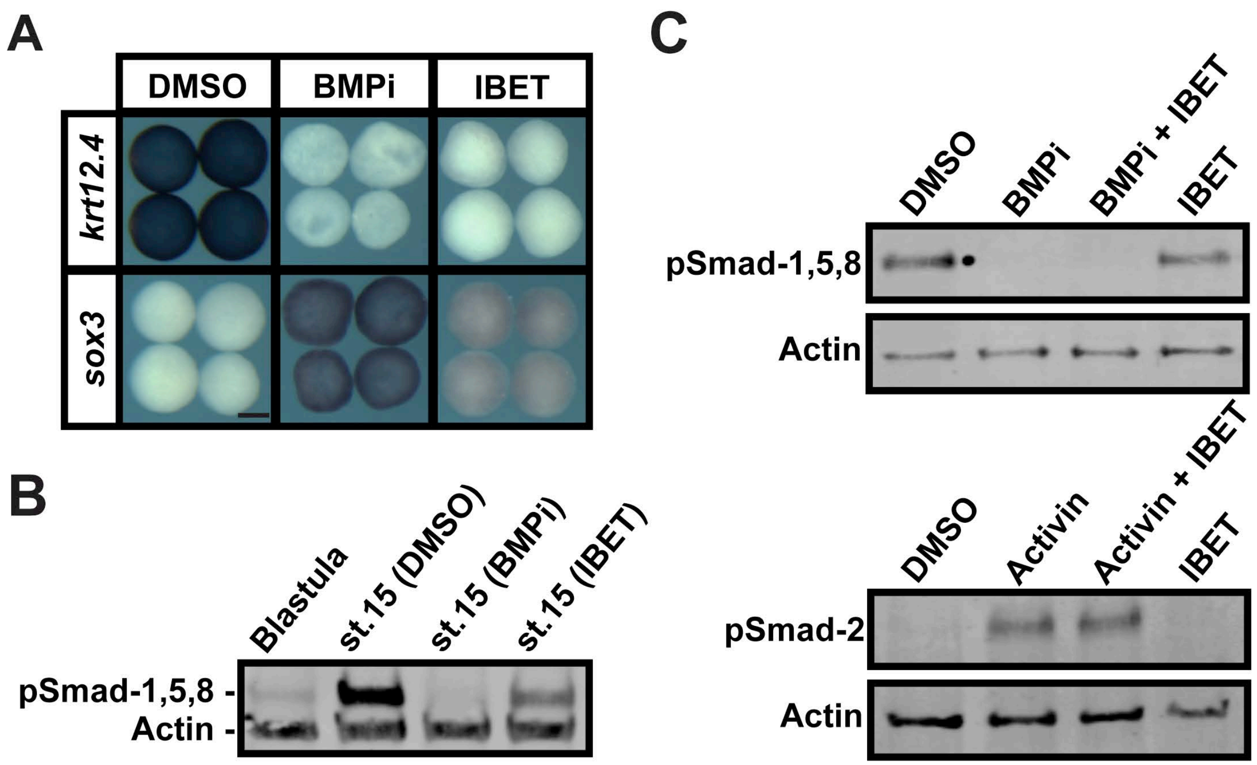

Fig. S9. (A)*In situ* hybridization examining the expression of *sox3* and *krt12.4* in explanted blastula caps treated with vehicle, BMPi (20 μM) or IBET (250 μM) from stage 9 to 15, and (B) Western blot analysis of blastula explants and those treated identically as in (A) probed with antibodies for pSmad-1,5,8 and actin. (C) Western blot analysis of stage 11 explants treated with vehicle, BMPi (20 μM) or activin, BMPi (20 μM) or activin plus IBET (250 μM), or IBET (250 μM) from blastula stage until collection probed with antibodies for either pSmad-1,5,8 or pSmad-2 and actin. Scale bars: 250 μm

Table S1. Genes significantly changed by TSA and IBET List of genes significantly altered in response to both TSA and IBET by category

Available for download at
https://journals.biologists.com/dev/article-lookup/doi/10.1242/dev.202990#supplementary-data
